# Brain aging rejuvenation factors in adults with genetic and sporadic neurodegenerative disease

**DOI:** 10.1093/braincomms/fcae432

**Published:** 2025-01-15

**Authors:** Kaitlin B Casaletto, Rowan Saloner, John Kornak, Adam M Staffaroni, Saul Villeda, Emily Paolillo, Anna M VandeBunte, Claire J Cadwallader, Argentina Lario Lago, Julia Webb, Coty Chen, Katya Rascovsky, Toji Miyagawa, Eliana Marisa Ramos, Joseph C Masdeu, Alexander Pantelyat, Maria Carmela Tartaglia, Andrea Bozoki, Peter S Pressman, Rosa Rademakers, Walter Kremers, Ryan Darby, Kyan Younes, Belen Pascual, Nupur Ghoshal, Maria Lapid, Ian R A Mackenzie, Jingyao Li, Ging-Yuek Robin Hsiung, Jacob N Hall, Maya V Yutsis, Irene Litvan, Victor W Henderson, Rajeev Sivasankaran, Katie Worringer, Kimiko Domoto-Reilly, Allison Snyder, Joseph Loureiro, Joel H Kramer, Hilary Heuer, Leah K Forsberg, Howard J Rosen, Bradley Boeve, Julio C Rojas, Adam L Boxer

**Affiliations:** Department of Neurology, Memory and Aging Center, University of California, San Francisco, CA 94158, USA; Department of Neurology, Memory and Aging Center, University of California, San Francisco, CA 94158, USA; Department of Epidemiology and Biostatistics, University of California, San Francisco, CA 94158, USA; Department of Neurology, Memory and Aging Center, University of California, San Francisco, CA 94158, USA; Department of Anatomy, University of California, San Francisco, CA 94143, USA; Department of Neurology, Memory and Aging Center, University of California, San Francisco, CA 94158, USA; Department of Neurology, Memory and Aging Center, University of California, San Francisco, CA 94158, USA; Department of Neurology, Memory and Aging Center, University of California, San Francisco, CA 94158, USA; Department of Neurology, Memory and Aging Center, University of California, San Francisco, CA 94158, USA; Department of Neurology, Memory and Aging Center, University of California, San Francisco, CA 94158, USA; Department of Neurology, Memory and Aging Center, University of California, San Francisco, CA 94158, USA; Department of Neurology and Penn Frontotemporal Degeneration Center, University of Pennsylvania, Philadelphia, PA 19104, USA; Department of Neurology, Mayo Clinic, Rochester, MN 55905, USA; Department of Neurology, University of California, Los Angeles, CA 90095, USA; Department of Neurology, Houston Methodist, Houston, TX 77030, USA; Department of Neurology, Johns Hopkins University, Baltimore, MD 21287, USA; Tanz Centre for Research in Neurodegenerative Diseases, Division of Neurology, University of Toronto, Toronto, ON M5S 3H2, Canada; Department of Neurology, University of North Carolina, Chapel Hill, NC 27599, USA; Department of Neurology, University of Colorado, Aurora, CO 80045, USA; Neurodegenerative Brain Diseases Group, VIB Center for Molecular Neurology, University of Antwerp-Biomedical sciences, Antwerp, 2610, Belgium; Department of Quantitative Health Sciences, Mayo Clinic, Rochester, MN 55905, USA; Department of Neurology, Vanderbilt University, Nashville, TN 37212, USA; Department of Neurology, Stanford University, Palo Alto, CA 94304, USA; Department of Neurology, Houston Methodist, Houston, TX 77030, USA; Departments of Neurology and Psychiatry, Washington University, St. Louis, MO 63110, USA; Department of Psychiatry and Psychology, Mayo Clinic, Rochester, MN 55905, USA; Department of Pathology, University of British Columbia, Vancouver, BC V6T 2B5, Canada; Novartis Institutes for BioMedical Research, Cambridge, MA 02139, USA; Department of Medicine, Division of Neurology, University of British Columbia, Vancouver, BC V6T 2B5, Canada; The Neurology Center of Southern California, Carlsbad, CA 92011, USA; Department of Neurology and Neurological Sciences, Stanford University, Palo Alto, CA 94304, USA; Department of Neurosciences, University of California, La Jolla, CA 92037, USA; Departments of Epidemiology and Population Health and of Neurology and Neurological Sciences, Stanford University, Palo Alto, CA 94304, USA; Novartis Institutes for BioMedical Research, Cambridge, MA 02139, USA; Novartis Institutes for BioMedical Research, Cambridge, MA 02139, USA; Department of Neurology, University of Washington, Seattle, WA 98195, USA; National Institute of Neurological Disorders and Stroke, National Institutes of Health, Bethesda, MD 20814, USA; Novartis Institutes for BioMedical Research, Cambridge, MA 02139, USA; Department of Neurology, Memory and Aging Center, University of California, San Francisco, CA 94158, USA; Department of Neurology, Memory and Aging Center, University of California, San Francisco, CA 94159, USA; Department of Neurology, Mayo Clinic, Rochester, MN 55905, USA; Department of Neurology, Memory and Aging Center, University of California, San Francisco, CA 94158, USA; Department of Neurology, Mayo Clinic, Rochester, MN 55905, USA; Department of Neurology, Memory and Aging Center, University of California, San Francisco, CA 94158, USA; Department of Neurology, Memory and Aging Center, University of California, San Francisco, CA 94158, USA

**Keywords:** frontotemporal dementia, Alzheimer’s disease, cerebrospinal fluid, biomarkers, heterochronic parabiosis

## Abstract

The largest risk factor for dementia is age. Heterochronic blood exchange studies have uncovered age-related blood factors that demonstrate ‘pro-aging’ or ‘pro-youthful’ effects on the mouse brain. The clinical relevance and combined effects of these factors for humans is unclear. We examined five previously identified brain rejuvenation factors in cerebrospinal fluid of adults with autosomal dominant forms of frontotemporal dementia and sporadic Alzheimer’s disease. Our frontotemporal dementia cohort included 100 observationally followed adults carrying autosomal dominant frontotemporal dementia mutations (M_age_ = 49.6; 50% female; 43% *C9orf72*, 24% *GRN*, 33% *MAPT*) and 62 non-carriers (M_age_ = 52.6; 45% female) with cerebrospinal fluid analysed on Somascan, and longitudinal (M_visits_ = 3 years, range 1–7 years) neuropsychological and functional assessments and plasma neurofilament light chain. Our Alzheimer’s disease cohort included 35 adults with sporadic Alzheimer’s disease (M_age_ = 69.4; 60% female) and 56 controls (M_age_ = 68.8, 50% female) who completed the same cerebrospinal fluid and clinical outcome measures cross-sectionally. Levels of C-C motif chemokine ligand 11, C-C motif chemokine ligand 2, beta-2-micorglobulin, bone gamma-carboxyglutamate protein (aka Osteocalcin) and colony stimulating factor 2 in cerebrospinal fluid were linearly combined into a composite score, with higher values reflecting ‘pro-youthful’ levels. In genetic frontotemporal dementia, higher baseline cerebrospinal fluid rejuvenation proteins predicted slower decline across cognitive, functional, and neurofilament light chain trajectories; estimates were similar across genotypes. In transdiagnostic analyses, higher cerebrospinal fluid rejuvenation proteins associated with better functional, cognitive, and neurofilament light chain outcomes in adults with sporadic Alzheimer’s disease. Proteins with pre-clinical evidence for brain rejuvenation show translational clinical relevance in adults with Alzheimer’s disease and related dementias and warrant further investigation.

## Teaser

In this translational study, Casaletto *et al*. examined proteins previously shown to demonstrate brain aging rejuvenation effects in mechanistic animal models. Rejuvenation proteins were measured in human cerebrospinal fluid and associated with better cognitive, functional and axonal degeneration outcomes in both familial forms of frontotemporal dementia and sporadic Alzheimer’s disease.

## Graphical Abstract

**Graphical Abstract:**
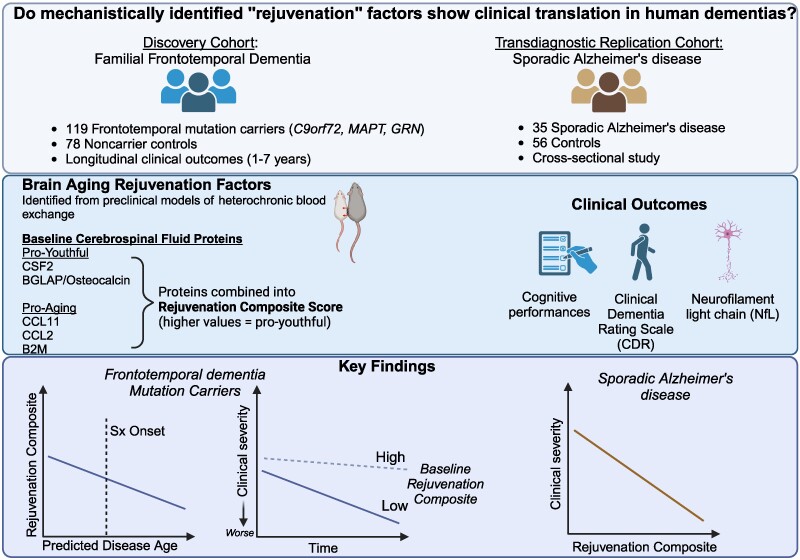

See Ng and Zetterberg (https://doi.org/10.1093/braincomms/fcae467) for a scientific commentary on this article.

## Introduction

Age is the largest risk factor for dementia, suggesting that fundamental aging biology may contribute to the pathophysiology of neurodegenerative diseases.^1,2^ The prevalence of dementia exponentially increases more than 6-fold with age, from 5% at ages 65–75 to >30% at ages 85 and older.^3^ Though proteinopathy specific drug therapies are emerging, multiple copathologies represent the vast majority of dementia cases with >75% of community-dwelling older adults showing at least two neuropathologies at autopsy.^4,5^ Even among dementias with earlier age at onset, copathologies contribute to disease onset and symptomatology in both frontotemporal dementia (FTD)^6,7^ and Alzheimer’s disease.^8^ Disentangling the shared neurobiological mechanisms of aging and neurodegenerative disease may represent a window into understanding transdiagnostic risk and identifying treatment and prevention targets for Alzheimer’s disease and Alzheimer’s disease-related dementias (ADRD).

Heterochronic blood experiments are the exchange of blood between differently aged animals to evaluate how age-dependent peripheral and circulating factors impact organ systems. These elegant experiments demonstrate that common aging factors can be isolated from the systemic milieu and causally shape the health of the aging brain.^9-11^ For instance, aged animals exposed to young animal plasma show ‘pro-youthful’ effects on brain (e.g. increased neurogenesis) and behavioural outcomes, while young animals exposed to aged animal plasma show ‘pro-aging’ effects on brain (e.g. decreased neurogenesis) and behavioural outcomes. To date, over a dozen individual blood factors have been identified from heterochronic blood experiments and mechanistically probed (e.g. via systemic injection, genetic knockout) in aged or young mice to recapitulate the pro-youthful or pro-aging effects on brain shown by heterochronic blood exchange, respectively.^9^ For instance, in a seminal study by Villeda and colleagues, young mice exposed to the systemic environment of aged mice either via conjoined circulatory systems or direct plasma exchange showed decreased synaptic plasticity and impaired fear conditioning and spatial learning and memory; in these experiments, higher plasma C-C motif chemokine ligand 11 (CCL11) associated with reduced neurogenesis and subsequent peripheral administration of CCL11 *in vivo* in young mice decreased adult neurogenesis and learning/memory performances(ref). These data suggest that individual circulating factors modulate brain aging trajectories; yet, human studies have yet to recapitulate these benefits. The major benefits of identifying brain biomarker and treatment targets from the systemic milieu (versus cerebrospinal fluid (CSF) targets) include circumventing issues related to blood–brain-barrier (BBB) access and increased patient scalability. Though several rejuvenation factors have begun to be measured in human samples,^10,12-16^ many have yet to be systematically tested for clinical relevance. Further, though heterochronic blood experiments are elegantly designed to identify and rigorously test causality of individual factors for brain aging, these factors have been examined in isolation to date, mainly in mouse models. Emerging human data suggest aging factors may interact to contribute to neurodegenerative risk.^16,17^ Given the complexity of the biology underlying brain aging and dementia risk, the simultaneous modulation of multiple targets may prove to be a more mechanistically sound and effective approach towards dementia prevention. Therefore, the next critical step to move promising targets identified from pre-clinical animal models into clinical trials is to evaluate their relevance more comprehensively in humans.

We cross-referenced the heterochronic blood experiment literature for targets that have been mechanistically shown to modulate brain aging^9^ and were quantified on the SomaScan assay.^18^ Blood is an optimal matrix to screen for targets with therapeutic potential for brain health.^19^ However, blood factors are influenced by many organ systems and may not directly reflect brain-related processes. Therefore, to more closely estimate how potential targets functioned in the context of the human central nervous system, we assayed CSF. To support biological plausibility for the heterochronic blood targets in CSF, we focused only on targets that may cross the BBB based on *in vivo* animal model data.^20-24^ We identified five proteins that met these criteria. Three ‘pro-aging’ factors: CC motif chemokine 11 (CCL11, aka eotaxin-1), CC motif chemokine 2 (CCL2, aka MCP-1), beta-2 microglobulin (B2 M) and two ‘pro-youthful’ factors: colony stimulating factor-2 (CSF2) and osteocalcin (BGLAP). Apart from BGLAP, which is primarily secreted by osteoblasts, all of the identified factors are directly synthesized by immune cells (e.g. monocytes/macrophages, T and B cells), as well as endothelial and epithelial cells and fibroblasts (i.e. CSF2, CCL11 and CCL2). Our goals were to (i) determine the translational relevance of these factors for clinical outcomes in human disease and (ii) test their combined (versus isolated) effects. To do so, we included a longitudinal discovery cohort of adults carrying pathogenic mutations for autosomal dominant FTD and non-carrier controls, and a cross-sectional transdiagnostic validation cohort of adults with sporadic Alzheimer’s disease and control participants. We examined how CSF levels of the five ‘rejuvenation proteins’ (combined and in isolation) associated with distinct clinical outcomes—objective cognitive performances, caregiver-rated functional decline, and fluid biomarker derived neurodegeneration (neurofilament light chain levels, NfL) in each cohort. Our data support the clinical relevance of mechanistically identified brain aging proteins across Alzheimer’s disease/ADRDs and underscore the utility of future work pursuing these targets, particularly exploring combination approaches for dementia prevention.

## Materials and methods

### Participant characteristics

Demographic and clinical data for both cohorts are presented in Table 1.

**Table 1:** Clinicodemographic characteristics of FTD and sporadic Alzheimer’s disease cohorts

	FTD pathogenic variant carriers (*n* = 119)	Non-carrier controls (*n* = 78)	*P*-value	Sporadic Alzheimer’s disease (*n* = 35)	Controls (*n* = 56)	*P*-value
Age	50.6 (13.1)	54.8 (15.8)	0.10	69.4 (9.7)	68.8 (8.6)	0.50
Education	15.4 (2.4)	16.5 (2.6)	0.036	16.3 (2.2)	16.9 (2.9)	0.45
Female sex (%, *n*)	52% (63)	56% (44)	0.74	60% (21)	50% (28)	0.50
Global cognition	−0.33 (0.94)	0.37 (0.53)	<0.001	−3.26 (2.12)	−0.02 (0.95)	<0.001
	(*n* = 101)	(*n* = 61)		(*n* = 30)	(*n* = 55)	
Genotype	41% (49)	–	–	–	–	−
C9orf72	28% (33)					
GRN	31% (37)					
MAPT						
Clinical dementia rating (CDR®) global score^a^ (median, IQR)	0 (0, 1)	0 (0, 0)	**–**	0.5 (0.5, 1)	0 (0,0)	−
	Range: 0 to 2	(*n* = 65)		(*n* = 34)	(*n* = 56)	
	(*n* = 119)					
SomaScan relative fluorescence units (CSF)^b^						
CCL2	9.36 (0.42)	9.24 (0.52)	0.12	254.13 (42.06)	263.42 (33.09)	0.27
CCL11	8.31 (0.39)	8.42 (0.39)	0.14	264.46 (38.92)	273.52 (38.96)	0.31
B2M	13.38 (0.20)	13.32 (0.19)	0.11	70149.4 (10020.75)	71261.8 (7736.05)	0.57
BGLAP	8.42 (0.19)	8.49 (0.19)	0.043	891.1 (231.43)	864.05 (208.83)	0.58
CSF2	6.810 (0.29)	6.814 (0.25)	0.93	67.90 (8.10)	72.47 (26.96)	0.37
Rejuvenation composite	−0.15 (0.99)	0.23 (0.97)	0.008	0.11 (0.45)	0.003 (0.50)	0.27
	Range: −2.57 to 2.18	Range: −1.74 to 3.49		Range: −0.64 to 1.03	Range: −1.21 to 1.71	
Neurofilament light chain (pg/mL) (median, IQR)	8.54 (4.82, 15.10)	5.62 (4.32, 8.24)	0.004	1070 (554.8, 1661.5)	731 (363.2, 988)	0.005
	(plasma, *n* = 91)	(plasma, *n* = 41)				
				(CSF, *n* = 16)	(CSF, *n* = 27)	

^a^CDR represents modified CDR® plus NACC-FTLD in FTD cohort.

^b^Relative Value Units based on SomaScan version 4.1 (FTD cohort) or version 4.0 (Alzheimer’s disease cohort). Sample sizes noted for any variables not available on the entire cohort.

#### FTD discovery cohort

Participants in the discovery cohort were drawn from the Advancing Research and Treatment for Frontotemporal Lobar Degeneration (ARTFL) and Longitudinal Evaluation of Familial Frontotemporal Dementia (LEFFTDS) Longitudinal FTD study (ALLFTD, ClinicalTrials.gov, NCT04363684) based in the United States and Canada. Participants included individuals carrying one of the three most common FTD pathogenic mutations (*n* = 119 total: 37 *MAPT,* 33 *GRN,* 49 *C9orf72*) and healthy control participants who did not carry a pathogenic mutation (*n* = 78) who completed lumbar puncture and CSF analysed on the SomaScan assay.^18^ Approximately half (*n* = 52) of pathogenic mutation carriers were asymptomatic at baseline (Global CDR® plus National Alzheimer’s Coordinating Center (NACC) FTLD = 0). ALLFTD is an ongoing longitudinal study with approximately annual visits. Participants completed a baseline visit and averaged 3.3 annual evaluations (total range 1–7 visits) during which comprehensive neuropsychological testing (baseline *n* = 184), caregiver-rated functional evaluations (baseline *n* = 112) and blood draws (baseline *n* = 132) with plasma analysed for NfL were completed. All genetic testing was completed in the same laboratory at the University of California, Los Angeles using standardized methods previously described.^25,26^

#### Alzheimer’s disease replication cohort

To test the transdiagnostic relevance of the identified rejuvenation factors, a cohort of 91 participants (35 Alzheimer’s disease and 56 clinically normal older adults) who completed lumbar puncture with CSF analysed on the SomaScan assay (version 4.1, 7k proteins)^27^ were selected from the Stanford University Alzheimer’s Disease Research Center (ADRC). All participants completed the same clinical outcomes as the discovery cohort, including cognitive testing, caregiver-based functional assessment, and CSF NfL quantification. Diagnostic classification was based on clinical research consensus criteria.^28^ Participant evaluations were cross-sectional in the replication cohort.

Participants in each cohort provided written informed consents and the study was approved by the local Institutional Review Boards.

### CSF rejuvenation protein quantification

Lumbar punctures were completed by a board-certified neurologist with CSF processed and stored following standard procedures.^17^ CSF was analysed on Somascan (SomaLogic, Inc.) using a proprietary version of the SomaScan proteomics platform (SomaLogic, Boulder, CO) that captured 4138 unique proteins for ALLFTD subjects or the 4.1 version platform that captured ∼7000 unique proteins for Stanford ADRC subjects.^18,27^ SomaLogic is a CLIA accredited laboratory. The Somascan platform uses aptamer technology (slow off-rate modified aptamer reagents, Somamers) to transform a protein signal into a nucleotide signal that can be quantified using relative florescent unit, which were normalized to scale. Somascan demonstrates high-precision detection (median coefficient of variance 5%) of even low abundance proteins (median 1 pM) with a large dynamic range (100 fM—1 mM).

### NfL quantification

NfL was quantified in plasma for ALLFTD and in CSF for Stanford ADRC participants. For both matrices, NfL concentrations were quantified in duplicate using the ultrasensitive HDX analyser by single molecule array (Simoa) technology (Quanterix, Billerica, MA) by investigators blinded to clinical group allocation.^29^ Samples with coefficients of variance >20% were excluded from analyses.

### Neuropsychological measures

Both the ALLFTD and Stanford ADRC participants completed a comprehensive neuropsychological battery, covering episodic memory, executive functions and language skills.^30,31^ We created sample-based z-scores on each individual cognitive measure, which were then averaged into domain-based composite scores. Episodic memory, executive functions and language domain composite scores were then averaged together to create a global cognitive performance composite. Global cognitive performance was examined as a primary clinical outcome of interest.

### Functional decline: clinical dementia rating scale

For both cohorts, informant-rated level of functional decline was measured via structured interview on the traditional clinical dementia rating scale (CDR**®)** (Stanford ADRC participants) or a validated, modified version of the CDR (CDR®+NACC FTLD) that is more appropriate for use in FTD spectrum patients (ALLFTD participants).^32^

In the traditional CDR**®** completed by Stanford participants, six domains of functional impairment (memory, orientation, judgement and problem solving, community affairs, home and hobbies and personal care) are rated on a 0 (absent) to 5 (severe) point scale. Higher scores indicate greater level of functional severity.

ALLFTD participants completed the Clinical Dementia Rating Instrument PLUS NACC Behaviour and Language Domains (CDR® plus NACC FTLD). The CDR®+NACC FTLD^25,32^ is similarly a marker of functional severity and includes ratings across six functional domains captured in the traditional CDR**®**, in addition to two domains specific to the core clinical features of FTLD: language and behaviour. Following a standardized algorithm,^25,32^ the eight domain scores were summed to create a global score (0–8), while each domain was scored on a scale from 0 to 3 and summed to create a more continuous measure of symptom severity (0–24) referred to as the sum of boxes (CDR®+NACC FTLD-SB).

### Latent disease age

Latent disease age was estimated for FTD mutation carriers based on recently developed disease progression models.^31^ Briefly, latent disease age is the estimated difference between an individuals’ chronological age and age of symptom onset (operationalized as CDR®+NACC FTLD-SB = 0.5). The disease progression model was estimated based on 20 measures previously shown to contribute to disease prognostication in FTD (e.g. all available NfL, imaging, neuropsychological performances, and clinical information). The estimate is positive for symptomatic cases and negative for pre-symptomatic cases. For symptomatic cases, initial estimates reflect clinician’s estimated time from symptom onset modelled with an error term. For asymptomatic cases, initial estimates reflect the difference between chronological age and mean age of genotype onset at the group level, modelled with an error term. Initial estimates were then submitted to Bayesian mixed effects framework leveraging all available clinical, neuropsychological, imaging and NfL data simultaneously to more precisely estimate latent disease age (see^31^ for additional details).

### Statistical analyses

#### Discovery cohort: longitudinal FTD analyses

We created a sample-based z-score on individual target protein levels and the five target proteins were linearly combined into a ‘rejuvenation composite’ score in which each protein was equally weighted. We examined the relationships between rejuvenation composite levels across mutation carriers versus non-carriers and with clinicodemographic features, including latent disease age, at baseline. Next, we evaluated the relationship between baseline levels of the rejuvenation composite and longitudinal trajectories of global cognitive performances, functional decline (CDR®+NACC FTLD) and plasma NfL by mutation carrier status via linear mixed effects (LME) models. LME models included an interaction term between baseline rejuvenation composite by time in study (in years) by mutation carrier status (yes/no), adjusting for baseline age, sex and education, and allowed for random (person-specific) intercepts and slopes. We estimated effect sizes between rejuvenation composite and cognitive, function and NfL outcomes in mutation carriers by calculating the difference in the predicted marginal mean from participants in the top versus bottom tertile of the rejuvenation composite over a 5-year interval; marginal mean differences and 95% CI reported. Given the possibility that disease severity may drive relationships, we conducted sensitivity analyses only including mutation carriers who were asymptomatic at baseline (*n* = 52; CDR®+NACC FTLD = 0). Additionally, we examined how relationships between our clinical outcomes and the rejuvenation composite differed by genotype. To do so, we evaluated the interaction of genotype on the relationship between rejuvenation composite and clinical trajectories. Lastly, within mutation carriers only, we probed the relative effect size of each individual protein within the rejuvenation composite to better understand the contribution of each of the five factors. To do so, we conducted parallel LMEs examining both (i) each individual protein and (ii) the rejuvenation composite removing each individual protein by time as a function of our three longitudinal outcomes (cognition, functioning and NfL).

#### Transdiagnostic relevance: cross-sectional Alzheimer’s disease analyses

To test the transdiagnostic relevance of these five rejuvenation factors, we reconstructed a ‘rejuvenation composite’ score in the Stanford ADRC cohort following the same method described above. This cohort was only evaluated cross-sectionally and included adults with Alzheimer’s disease and typically aging adults. First, we examined associations between the rejuvenation composite and clinicodemographic information using Pearson correlation and *t*-tests, as appropriate. Next, we conducted regression models examining the interaction between rejuvenation composite levels and diagnosis on the same three clinical outcomes—global cognition, functional impairment and CSF NfL levels.

## Results

### CSF rejuvenation proteins and baseline clinicodemographic characteristics in FTD

**Figure 1:**
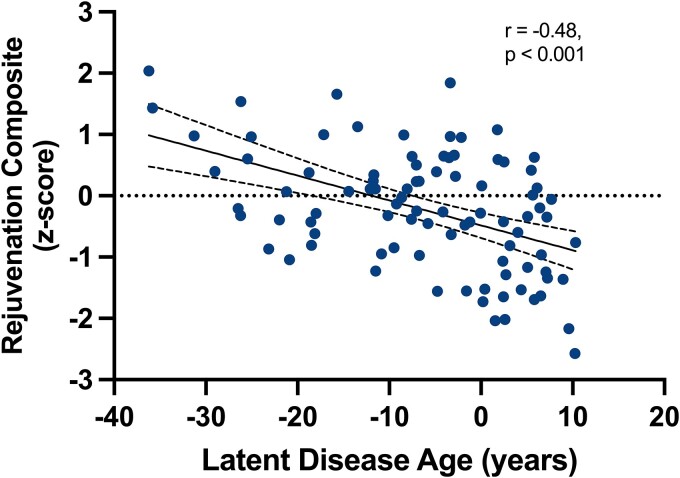
**Adults carrying autosomal dominant genetic mutations for FTD who are closer to estimated disease onset show lower levels of CSF rejuvenation protein levels.** Error bars represent 95% CI. Pearson’s correlational analyses displayed in which each data point represents a participant (*n* = 119).

In FTD mutation carriers and non-carriers combined, CSF levels of the five targeted factors showed small to minimal intra-analyte correlations (Supplementary Table 1), suggesting only modest overlap in the biological processes represented. Given combining factors is novel and there is no evidence to support relative importance of one factor over another, the five CSF factors were linearly combined into a composite score in which each protein was equally weighted. Higher values reflected more ‘pro-youthful’ protein levels. Higher rejuvenation composite levels modestly associated with younger age (r = −0.16, 95% CI −0.29 to −0.02, *P* = 0.03), but showed minimal associations with education (r = 0.05, 95% CI −0.09 to 0.19 *P* = 0.45) or sex (M_men_ = 0.02 (SD = 0.98) versus M_women_ = −0.02 (SD = 1.0), *P* = 0.78). CSF rejuvenation composite levels were statistically significantly lower in mutation carriers compared to non-carriers at baseline (M_carriers_ = −0.15 (SD = 1.0) versus M_non-carriers_ = 0.23 (SD = 0.98), *P* = 0.008; Table 1). We next examined associations between CSF rejuvenation composite and estimated disease age in pathogenic mutation carriers. Disease age was estimated using a recently developed algorithm from the ALLFTD Study, which forecasts pre-symptomatic mutation carriers’ proximity to symptom onset based on joint modelling of the best known clinical and biomarker measures of FTD severity.^31^ In mutation carriers, cross-sectional analyses showed higher levels of the CSF rejuvenation composite associated with lower estimated disease age at baseline (*r* = 0.48, 95% CI −0.62 to −0.30, *P* < 0.001; Fig. 1). In other words, FTD mutation carriers who are closer to estimated disease onset show lower levels of CSF rejuvenation protein levels.

### Higher baseline CSF rejuvenation composite levels associate with slower clinical progression in FTD

**Figure 2:**
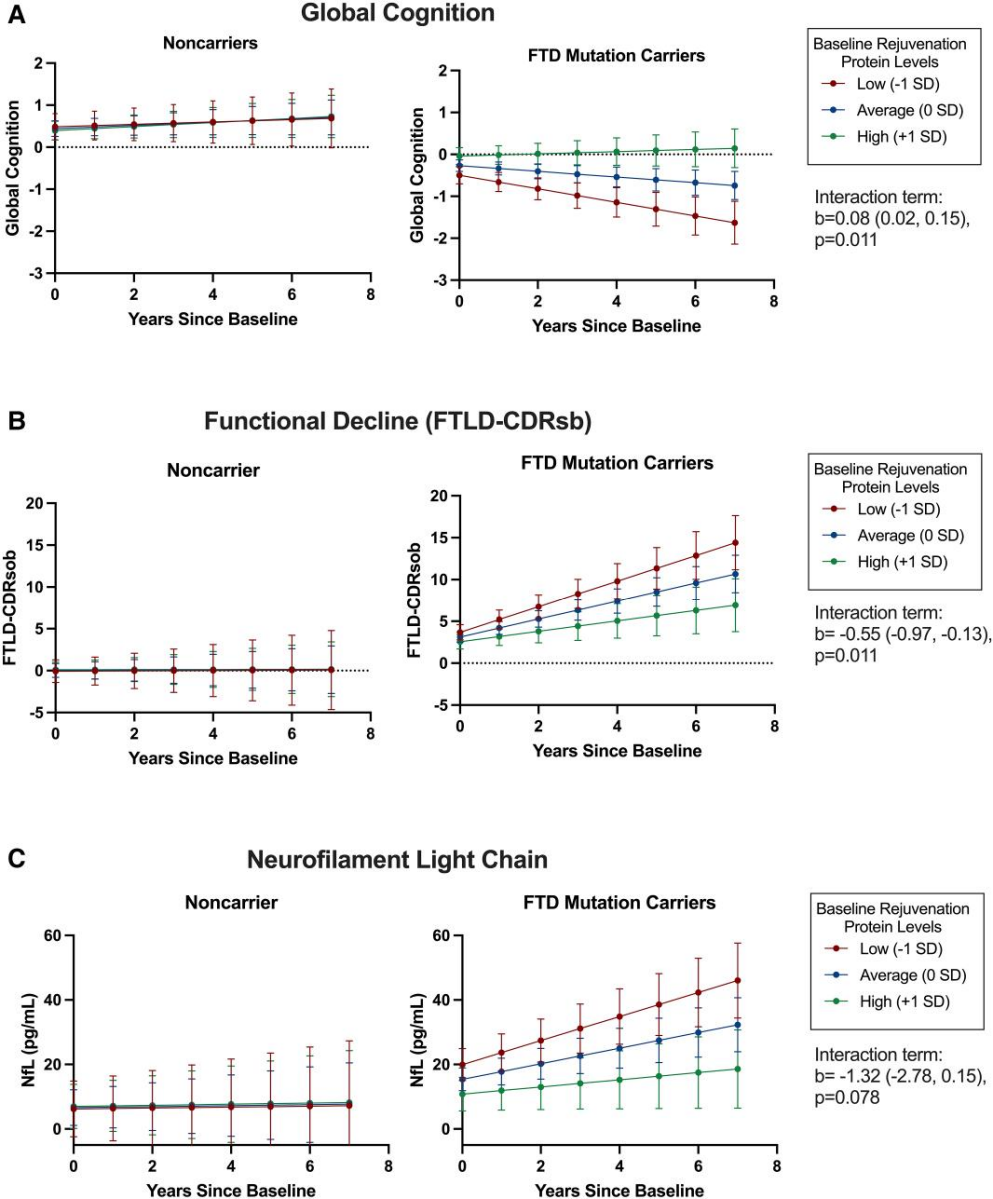
**Adults carrying autosomal dominant genetic mutations for FTD with higher baseline CSF rejuvenation protein levels show slower (A) cognitive, (B) functional and (C) neurodegenerative decline over time.** Error bars represent 95% CI; SD, standard deviation; FTLD-CDRsb, frontotemporal lobar degeneration CDR scale sum of boxes; NfL, neurofilament light chain. LMEs models are displayed covarying for baseline age, sex and education for cognitive (*n* = 162), functional (*n* = 184) and NfL (*n* = 132) trajectories.

**Figure 3:**
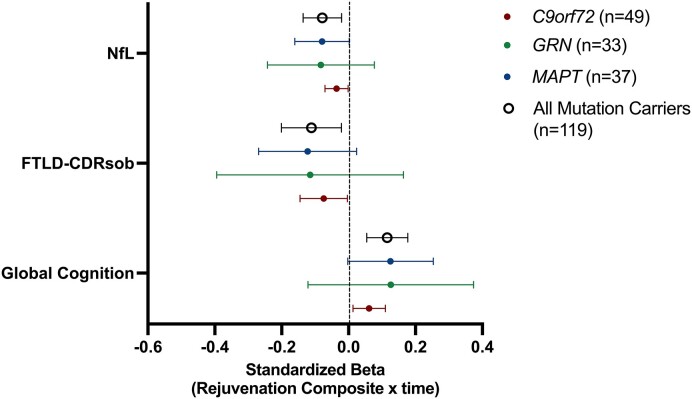
**Associations between CSF rejuvenation protein levels and clinical trajectories are similar across FTD genotypes.** Effect sizes are standardized betas with 95% CI; FTLD-CDRsb, frontotemporal lobar degeneration CDR scale sum of boxes; NfL, neurofilament light chain. Effect sizes from LMEs models are displayed covarying for baseline age, sex and education for cognitive (*n* = 162), functional (*n* = 184) and NfL (*n* = 132) trajectories.

Next, we tested how baseline levels of the CSF rejuvenation composite moderated longitudinal clinical trajectories in FTD mutation carriers compared to non-carriers via linear mixed-effects models. As shown in Table 2, baseline CSF rejuvenation composite levels demonstrated an interaction with carrier status (yes/no) on clinical progression over time, adjusting for baseline age, sex and education. FTD mutation carriers with higher baseline CSF rejuvenation composite levels demonstrated slower global cognitive and functional declines, and slower plasma NfL increases which approached significance (*P* = 0.078), relationships that were not evident in clinically normal non-carrier adults (Table 2; Fig. 2; Supplementary Fig. 1). Compared to carriers with low rejuvenation protein levels (bottom tertile), higher baseline rejuvenation protein levels (top tertile) were associated with ∼8.0-times slower cognitive (Low Rejuvenation_Marginal Mean_ = −0.97, 95% CI −1.27 to 0.66 versus High Rejuvenation_Marginal Mean_ = 0.12, 95% CI −0.016 to 0.40), 2.6-times slower functional (Low Rejuvenation_Marginal Mean_ = 7.64, 95% CI 5.53 to 9.75 versus High Rejuvenation_Marginal Mean_ = 2.89, 95% CI 0.78 to 5.02) and 3.4-times slower NfL increases (Low Rejuvenation_Marginal Mean_ = 18.62, 95% CI 12.95 to 24.29 versus High Rejuvenation_Marginal Mean_ = 5.51, 95% CI −0.41 to 11.43) over an estimated 5-years. Notably, when only including mutation carriers who were clinically asymptomatic at baseline (*n* = 52; CDR®+NACC FTLD = 0), all models were statistically significant with similar effect sizes (Supplementary Table 2). Further, examining individual cognitive domains within FTD mutation carriers only, higher baseline rejuvenation protein levels significantly associated with slowed declines across all domains examined, including episodic memory, executive functioning and language (all *P*-values < 0.009). Additionally, there was no statistically significant interaction with genotype suggesting that the effect sizes of the relationship between baseline CSF rejuvenation composite levels on clinical outcomes did not differ across *C9orf72*, *MAPT* or *GRN* mutation carriers (Fig. 3).

**Table 2:** Mixed effects models demonstrating the relationship between baseline CSF rejuvenation composite with longitudinal cognitive, functional and NfL progression in FTD variant carriers and non-carrier controls

	Global cognition		Functional decline (NACC-FTLD-CDR®sb)		NfL (plasma)	
	*N* = 162 (153 observations) # Visits average = 3.3 (range 1–7)		*N* = 184 (625 observations) # Visits average 3.4 (range 1–7)		*N* = 132 (372 observations) # Visits average 2.8 (range 1–4)	
	Beta (95% CI)	*P*-value	Beta (95% CI)	*P*-value	Beta (95% CI)	*P*-value
Baseline age	−0.01 (−0.02, −0.005)	0.001	0.04 (0.009, 0.08)	0.014	0.25 (0.08, 0.43)	0.003
Sex	−0.16 (−0.36, 0.05)	0.14	−0.07 (−1.02, 0.88)	0.88	2.18 (−1.95, 6.30)	0.30
Education	0.14 (0.10, 0.19)	<0.001	−0.16 (−0.35, 0.04)	0.12	0.04 (−0.84, 0.92)	0.93
Baseline CSF rejuvenation composite	−0.46 (−0.24, 0.15)	0.65	−0.13 (−0.98, 0.73)	0.77	0.40 (−5.08, 5.87)	0.89
Time (years)	0.038 (−0.007, 0.08)	0.096	0.08 (−0.22, 0.38)	0.60	0.15 (−1.01, 1.32)	0.80
Carrier status (y/*n*)	0.01 (−0.04, 0.06)	0.38	3.69 (2.57, 4.81)	<0.001	8.60 (2.03, 15.2)	0.01
(Baseline CSF rejuvenation composite)* (time)	0.01 (−0.04, 0.06)	0.71	0.89 (0.50, 1.29)	<0.001	0.01 (−1.22, 1.25)	0.98
(Time)* (carrier status)	−0.11 (−0.16, −0.05)	<0.001	1.10 (0.70, 1.50)	<0.001	2.26 (0.86, 3.67)	0.002
(Baseline CSF Rejuvenation Composite)* (Carrier status)	0.27 (0.03, 0.52)	0.03	−0.57 (−1.66, 0.53)	0.31	−4.98 (−11.54, 1.57)	0.14
(Baseline CSF rejuvenation composite)* (time)* (carrier status)	0.08 (0.02, 0.15)	0.011	−0.55 (−0.97, −0.13)	0.01	−1.32 (−2.78, 0.15)	0.078

Carrier status: noncarrier = 0; mutation carrier = 1.

**Figure 4:**
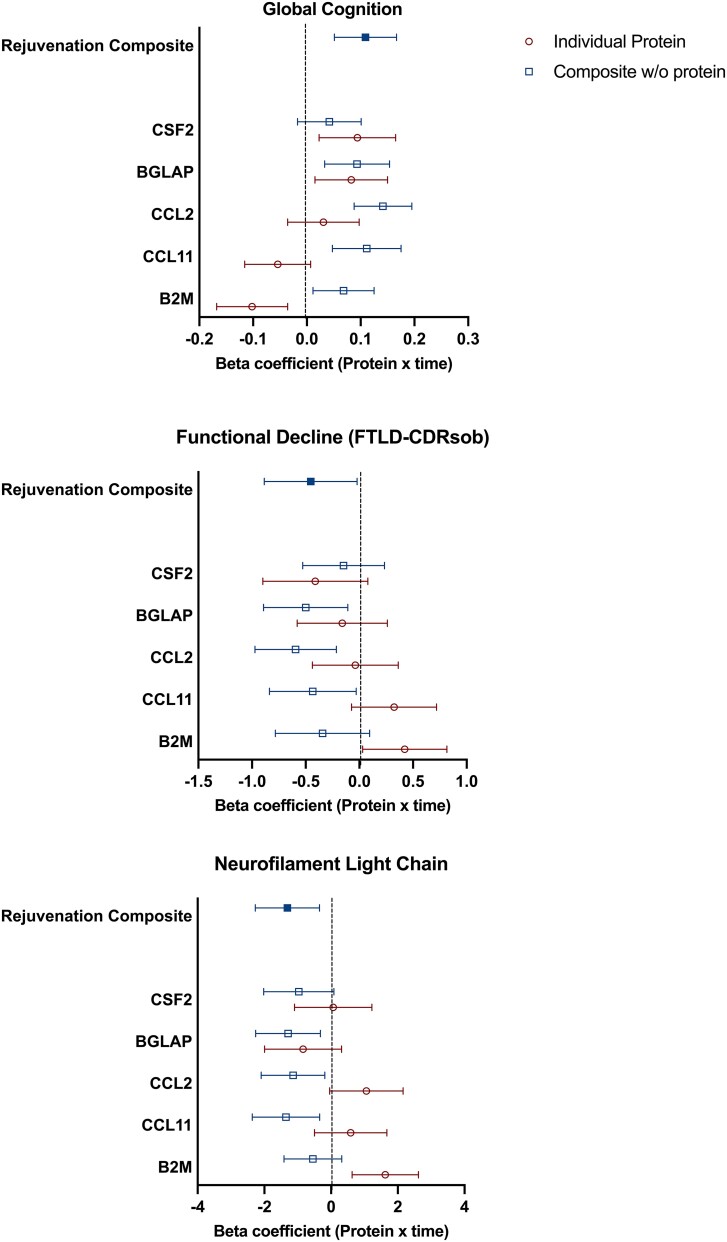
**Associations between CSF rejuvenation protein levels and clinical outcomes are most consistent using a composite approach.** Filled blue squares represent the effect size between the composite and clinical trajectories. Open red circles represent effect size estimates between individual protein levels and clinical trajectories. Open blue squares represent the effect size of the composite with clinical trajectories when removing the indicated protein. Effect sizes are standardized betas with 95% CI; FTLD-CDRsb, frontotemporal lobar degeneration CDR scale sum of boxes. Effect sizes from LMEs models are displayed covarying for baseline age, sex and education for cognitive (*n* = 162), functional (*n* = 184) and NfL (*n* = 132) trajectories.

Given, we only observed a meaningful relationship between CSF rejuvenation composite levels and clinical outcomes in mutation carriers, we next aimed to determine which, if any, of the five factors may be individually driving our findings. To test this, we examined how the relationship between the CSF rejuvenation composite and clinical outcomes changed when systematically removing one factor at a time and recalculating the standardized composite. Figure 4 shows that the relationship between the rejuvenation composite and clinical outcomes did not substantially change when removing individual proteins one at a time. Lastly, we examined the individual relationships between each of the five factors and our clinical outcomes to evaluate their independent importance. As per Fig. 4, baseline levels of BGLAP, CCL11 and B2 M showed the most consistent associations with longitudinal progression on all three clinical outcomes.

### Transdiagnostic relevance: higher CSF rejuvenation composite associates with less clinical severity in sporadic Alzheimer’s disease

**Figure 5:**
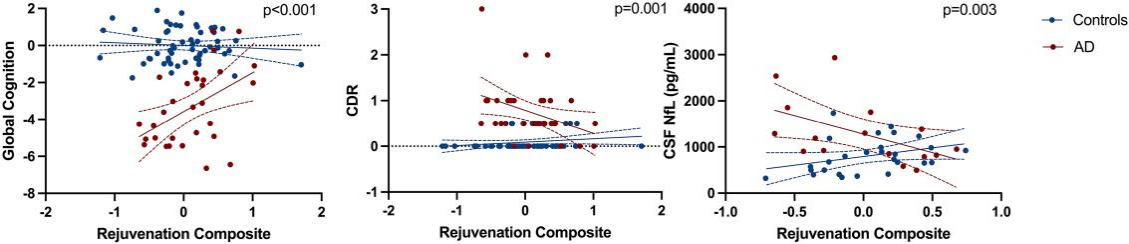
**Higher CSF rejuvenation protein levels associate with better clinical outcomes in adults with sporadic Alzheimer’s disease.**  *P*-value for interaction presented. Error bars represent 95% CI; CDR, clinical dementia rating scale global score; CSF, cerebrospinal fluid; NfL, neurofilament light chain. Multi-variable regression models were conducted adjusting for age, sex and education and scatterplots shown in which each dot represents a participant (*n* = 91).

In a cross-sectional cohort of controls and adults with sporadic Alzheimer’s disease, there similarly was an interaction between CSF rejuvenation composite levels and diagnosis on all three clinical outcomes, adjusting for age, sex and education. Higher CSF rejuvenation composite levels were associated with better cognitive performances and functional status, and lower CSF NfL (*n* = 43) among individuals with Alzheimer’s disease; these relationships were significantly attenuated in clinically normal adults (Fig. 5; Supplementary Table 3). These data recapitulate the longitudinal neuroprotective associations observed in the FTD mutation carriers. When examining specific cognitive domains within Alzheimer’s disease participants, higher rejuvenation protein levels associated with better episodic memory performances (B = 0.55, *P* = 0.003), but did not reach statistical significance for language or executive functioning performances (*P*-values >0.50).

Again, given the observed relationships were only evident in adults with Alzheimer’s disease, we further examined how individual (versus composite) rejuvenation proteins contributed to clinical outcomes only among the Alzheimer’s disease participants (*n* = 35); analyses were only conducted examining cognitive performance and functional outcomes given limited sample size in Alzheimer’s disease participants with CSF NfL (*n* = 14). When examined individually, there was only a statistically significant association between lower CSF B2 M and better global cognitive performances (see Supplementary Fig. 2). B2 M also approached statistical significance for functional outcomes (*P* = 0.09) and CCL2 approached statistical significance for both cognitive (*P* = 0.052) and functional (*P* = 0.08) outcomes. CCL11, CSF2, and BGLAP did not show strong individual effects.

## Discussion

We demonstrate that systemic factors shown to modulate brain aging trajectories in mice associate with a range of clinical progression indicators in CSF of adults with both genetic and sporadic forms of multiple Alzheimer’s disease/ADRDs across distinct cohorts. CSF reflecting lower levels of three ‘pro-aging’ factors (CCL2, CCL11 and B2 M) and higher levels of two ‘pro-youthful’ factors (CSF2 and BGLAP) associated with slower cognitive, functional and neuroaxonal declines in adults with autosomal dominant forms of FTD and sporadic Alzheimer’s disease. Protective relationships were similarly observed across proteinopathies, including FTD TDP-43 (*C9orf72* and *GRN*), FTD tauopathy (*MAPT*), and Alzheimer’s disease, suggesting transdiagnostic relevance. Although not statistically significant in control subjects, we did observe protective associations between CSF rejuvenation factors and clinical outcomes among clinically asymptomatic adults carrying FTD mutations. These findings suggest the identified factors may become most relevant in the context of elevated disease risk, perhaps before clinical manifestation, but not in typical brain aging. Additionally, we show utility of combining multiple rejuvenation factors, which may reflect a more biologically robust approach to understanding and treating brain aging.^16^ Given these factors have been identified from the systemic milieu, cross the BBB, and are beginning to show clinical importance across dementias, they may be valuable targets for transdiagnostic biomarker and treatment approaches. Copathology contributes to disease manifestation and is the norm rather than the exception in both FTD and Alzheimer’s disease.^4-7^ Rejuvenation factors that capture shared Alzheimer’s disease/ADRD risk may therefore be paired with proteinopathy-specific biomarkers to more precisely prognosticate disease progression and/or be utilized in combination therapies with proteinopathy-lowering drugs to mitigate clinical manifestation of disease biology.

Our data also support the notion that common aging biology may underlie risk and resilience across Alzheimer’s disease/ADRDs. Disentangling the fundamental neurobiological mechanisms of aging will therefore support understanding of how diseases of mid to late life emerge. Four out of the five identified factors are known to be directly involved in immune functioning: CSF2 stimulates stem cell production of monocytes/granulocytes, CCL11 and CCL2 are chemokines closely clustered on chromosome 17, and B2 M is a molecular component of the MHC class I complex. Consistent with the broader heterochronic blood experiment literature and increasing genome wide association studies of FTD and Alzheimer’s disease,^33-36^ these data underscore the critical role of both innate and adaptive immune processes in shaping neurodegenerative risk. Regarding the role of adaptive immunity, it is notable that B2 M alone demonstrated some of the most consistent associations with all clinical outcomes and across disease cohorts, suggesting regulation of canonical antigen-presenting processes may be particularly relevant in dementia risk and/or prevention. Consistent with our models in Alzheimer’s disease participants, B2 M is also directly implicated in pathological amyloid accumulation, including development of systemic amyloidosis,^37,38^ is a component of the beta-amyloid plaque core, and promotes beta-amyloid aggregation and neurotoxicity in models of Alzheimer’s disease.^39^ To our knowledge, these are among the first data showing a role for B2 M in FTD suggesting that its neurotoxicity may extend beyond beta-amyloid. The immune system is directly modulated by *GRN* and *C9orf72*, and *MAPT* clinical onset is influenced by levels of inflammation.^34,40,41^ Our data further highlight the neuroimmune axis in FTD, specifically expanding a possible role for B2 M in the clinical manifestations of TDP-43 and primary tauopathies.

Supporting a therapeutic role for these factors, there was a recent Phase II double-blinded, randomized controlled trial targeting CSF2 (aka, GM-CSF) in Alzheimer’s disease patients via brief subcutaneous sargramostim treatment (5 days/week for 3-weeks with 90-day follow-up).^42^ Sargramostim is a repurposed medication used to stimulate white blood cells production in bone marrow. Alzheimer’s disease participants did not show any serious adverse effects (primary endpoint), and showed improvements in MMSE and plasma amyloid-beta, total tau and UCHL1 (secondary endpoints), though changes on other clinical outcomes were more variable (e.g. ADAS-cognition and amyloid PET).^42^ Another Phase II trial (NCT04902703) is now ongoing to evaluate safety and efficacy longer-term sargramostim treatment in Alzheimer’s disease. Our data underscore a protective relationship between CSF2 and Alzheimer’s disease related outcomes, and further suggest that CSF2 may also have relevance in FTD spectrum disorders.

In contrast with the other four immune related factors, BGLAP (aka and osteocalcin) is a bone-derived energy regulation factor involved in insulin synthesis. BGLAP has been shown to decrease in adulthood across species, crosses the BBB, and binds to brain stem (ventral tegmentum area and mid/dorsal raphe) and hippocampal regions.^20^ Pre-clinical studies suggest BGLAP can promote vesicular transport of brain derived neurotrophic factor, increase monoamine synthesis and inhibit GABA synthesis subsequently promoting learning and memory and preventing anxiety-like behaviours. Increasing evidence has identified a bone-to-brain axis^43,44^ underscoring that more a complete understanding of brain aging and dementia risk necessitates examination of factors from peripheral organ systems.

Of note, we did not observe statistically significant associations between the CSF rejuvenation composite and clinical outcomes in cognitively normal adults, which were unexpected. However, the average age of our control subjects was relatively young (50s–60 s) across cohorts. This parallels some animal studies in which systemic rejuvenation manipulation only modulates brain and behavioural outcomes when administered in the oldest (≥12 months) but not younger (e.g. 3 months) adult mice.^45,46^ Taken together, this suggests that modulation of these factors either does not meaningfully impact brain aging until a threshold of disease or pathology is reached, and/or these factors reflect a compensatory and modifiable response to disease pathology. These findings may also suggest that neurodegenerative diseases represents a form of ‘accelerated’ brain aging per these biomarkers that is not observed in our typically aging cohorts. Specificity to disease states (versus altering typical aging neurobiology) is a desirable quality for a treatment target; a deeper understanding of how, when, and in whom these rejuvenation factors may show clinical relevance is needed.

Although these are the first data combining and directly translating brain rejuvenation factors into multiple cohorts of adults with Alzheimer’s disease/ADRDs, our study has several limitations. Most notably, these are observational data without autopsy confirmation of neurodegenerative aetiology. Although we found protective relationships on longitudinal clinical progression and in adults who are asymptomatic but at high genetic risk (pre-clinical disease), we cannot determine directionality of relationships from our study design. Additionally, although over a dozen systemic brain rejuvenation factors have been identified in animal literature,^9^ we limited examination to only those factors quantified via the SomaScan assay utilized in both cohorts. Although the Somascan assay only captures a fraction of the CSF proteome, it is compelling that different version of the SomaScan assay (versions 3.1 and 4.0) were used across the two cohorts and showed consistency in relationships, supporting utility of aptamer-based platforms for proteomic capture. Nonetheless, replication of our findings in independent neurodegenerative disease cohorts and prospective quantification of these five and additional factors identified in the heterochronic blood experiment literature via alterative quantification methods (e.g. ELISA) are needed to support convergence. Lastly, although we evaluated patients with both sporadic and genetic forms of dementia with a range of proteinopathies (primary and secondary tauopathies, TDP-43), it would be highly relevant to evaluate other common brain diseases associated with aging to determine the range of possible clinical utility (e.g. alpha-synuclein and cerebrovascular disease).

Taken together, our findings suggest that mechanistically identified systemic brain rejuvenation factors reflect a common biology of aging relevant for slowed clinical progression across neurodegenerative aetiologies in humans. When tested individually in mouse and *in vitro* models, each of the five factors modulate brain aging, yet our data suggest protective effects may be most robustly observed when factors are considered together. Although proteinopathy-specific dementia treatments are needed, multiple neuropathologies are evident in the vast majority of older adult brains.^4-7^ Combination treatments simultaneously targeting disease-specific and orthogonal brain aging targets represent complementary approaches for tackling dementia prevention.

## Supplementary Material

fcae432_Supplementary_Data

## Data Availability

The datasets analysed for the current study reflect collaborative efforts of two research studies: ALLFTD and Stanford ADRC. Each study provides clinical data access based on established policies for data use: processes for request are available for review at allftd.org/data for ALLFTD data and by contacting Stanford ADRC administrative team. Certain data elements from both consortia (e.g. raw MRI images) may be restricted due to the potential for identifiability in the context of the sensitive nature of the genetic data.
