# Correction to: Brain aging rejuvenation factors in adults with genetic and sporadic neurodegenerative disease

**DOI:** 10.1093/braincomms/fcaf058

**Published:** 2025-02-07

This is a correction to: Kaitlin B Casaletto, Rowan Saloner, John Kornak, Adam M Staffaroni, Saul Villeda, Emily Paolillo, Anna M VandeBunte, Claire J Cadwallader, Argentina Lario Lago, Julia Webb, Coty Chen, Katya Rascovsky, Toji Miyagawa, Eliana Marisa Ramos, Joseph C Masdeu, Alexander Pantelyat, Maria Carmela Tartaglia, Andrea Bozoki, Peter S Pressman, Rosa Rademakers, Walter Kremers, Ryan Darby, Kyan Younes, Belen Pascual, Nupur Ghoshal, Maria Lapid, Ian R A Mackenzie, Jingyao Li, Ging-Yuek Robin Hsiung, Jacob N Hall, Maya V Yutsis, Irene Litvan, Victor W Henderson, Rajeev Sivasankaran, Katie Worringer, Kimiko Domoto-Reilly, Allison Snyder, Joseph Loureiro, Joel H Kramer, Hilary Heuer, Leah K Forsberg, Howard J Rosen, Bradley Boeve, Julio C Rojas, Adam L Boxer, on behalf of the ALLFTD Consortium, Brain aging rejuvenation factors in adults with genetic and sporadic neurodegenerative disease, *Brain Communications*, Volume 7, Issue 1, 2025, https://doi.org/10.1093/braincomms/fcae432

In the originally published version of the manuscript there was a typographical error in the last name of the 37th author. Their name has been emended in the article to read: “Allison Snyder”.

