# Supplementary material for: Brain aging rejuvenation factors in adults with genetic and sporadic neurodegenerative disease
Source: Brain Commun. 2025 Jan 15;7(1):fcae432. doi: 10.1093/braincomms/fcae432 (PMC11734525; doi:10.1093/braincomms/fcae432)

Supplementary Materials.

Supplementary Table 1. Intra-analyte Pearson correlations in FTD cohort.

	**CCL2**	**CCL11**	**B2M**	**BGLAP**
**CCL2**	--			
**CCL11**	0.107	--		
**B2M**	0.265	0.024	--	
**BGLAP**	-0.281	0.155*	-0.019	--
**CSF2**	0.121	0.008	0.132	-0.022

*p<0.05.

Supplementary Table 2. Mixed effects models demonstrating the relationship between baseline CSF Rejuvenation Composite and longitudinal cognitive, functional, and NfL progression in FTD variant carriers who were asymptomatic (CDR®NACC-FTLD=0) at baseline.

	**Global Cognition**		**Functional decline (NACC-FTLD-CDRsb)**		**NfL (plasma)**	
	N=52 (216 observations)<br># Visits average = 4.2 (range 1-7)		N=52 (234 observations)<br># Visits average = 4.3 (range 1-7)		N=52 (164 observations)<br># Visits average 3.2 (range 1-4)	
	beta (95% CI)	p-value	beta (95% CI)	p-value	beta (95% CI)	p-value
Baseline age	-0.01 (-0.02, 0.002)	0.096	0.002 (-0.003, 0.008)	0.35	0.13 (0.07, 0.18)	<0.001
Sex	-0.02 (-0.29, 0.26)	0.89	0.04 (-0.09, 0.17)	0.51	0.17 (-1.11, 1.44)	0.80
Education	0.14 (0.08, 0.21)	<0.001	-0.008 (-0.04, 0.02)	0.61	0.28 (-0.01, 0.57)	0.06
Baseline CSF Rejuvenation Composite	-0.004 (-0.17, 0.16)	0.97	0.11 (0.02, 0.19)	0.01	-0.88 (-1.67, -0.09)	0.03
Time (years)	0.025 (-0.007, 0.06)	0.13	0.22 (0.04, 0.40)	0.02	0.75 (0.39, 1.10)	<0.001
(Baseline CSF Rejuvenation Composite)* (Time)	0.06 (0.02, 0.09)	0.001	-0.28 (-0.47, -0.08)	0.005	-0.43 (-0.80, -0.06)	0.02

Supplementary Table 3. Cross-sectional regression transdiagnostic models demonstrating the relationship between cerebrospinal fluid (CSF) Rejuvenation Composite on cognitive, functional, and NfL levels in adults with sporadic Alzheimer's disease (AD) and controls.

	**Global Cognition**		**Functional decline (Clinical Dementia Rating Scale)**		**NfL (CSF)**	
	N=85		N=90		N=43	
	beta (95% CI)	p-value	beta (95% CI)	p-value	beta (95% CI)	p-value
Age	-0.02 (-0.06, 0.01)	0.17	-0.003 (-0.012, 0.007)	0.60	-5.07 (-27.9, 17.8)	0.66
Sex (REF: female)	-0.17 (-0.46, 0.11)	0.23	0.076 (-0.011, 0.16)	0.09	19.54 (-137.3, 176.4)	0.80
Education	0.14 (0.03, 0.25)	0.01	0.003 (-0.03, 0.04)	0.88	62.95 (-25.3, 151.2)	0.16
CSF Rejuvenation Composite	0.92 (0.28, 1.57)	0.006	-0.21 (-0.40, -0.01)	0.036	-241.11 (0646.2, 164.0)	0.24
Diagnostic group (REF: AD)	-1.68 (-1.97, -1.39)	<0.001	0.33 (0.25, 0.42)	<0.001	279.55 (113.0, 446.1)	0.002
(CSF Rejuvenation Composite)*(Diagnostic group)	-1.18 (0.57, 1.80)	0.0003	-0.31 (-0.49, -0.12)	0.001	-633.18 (-228.9, -0.43)	0.003

Supplementary Figure 1. Spaghetti plot demonstrating individual trajectories of clinical outcomes by baseline CSF rejuvenation protein levels in frontotemporal dementia (FTD) mutation carriers and noncarriers. CDR = Clinical Dementia Rating Scale global score. FTLD-CDRsb = Frontotemporal lobar degeneration Clinical Dementia Rating Scale sum of boxes; NfL = Neurofilament light chain. Linear mixed effects models are displayed covarying for baseline age, sex, and education for cognitive (n=162), functional (n=184), and NfL (n=132) trajectories. These plots recapitulate the primary linear mixed effects models evaluating the relationship between baseline CSF rejuvenation protein levels and longitudinal outcomes over the study period (Clinical outcomes ~ Baseline Rejuvenation Composite * time + baseline age + sex + education) but visualizes individual trajectories instead of group level estimations that are shown in Figure 2.

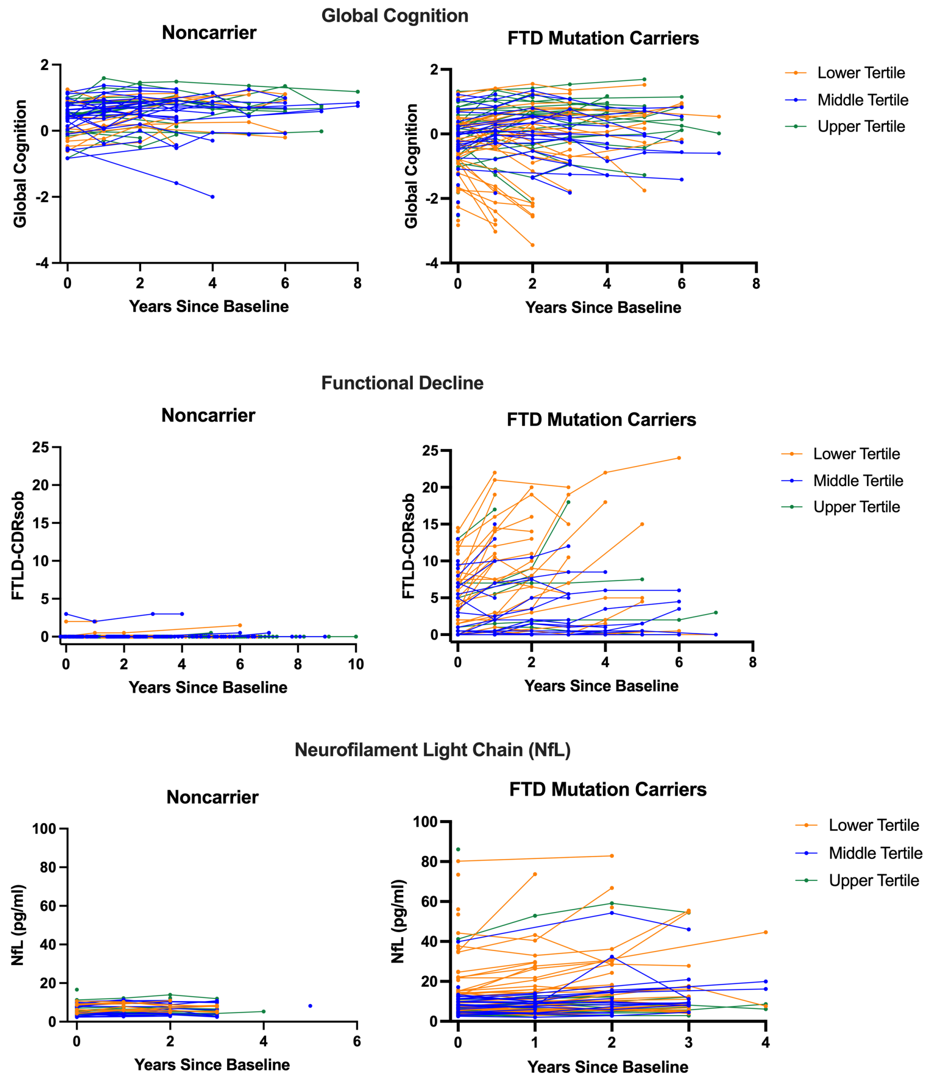

Supplementary Figure 2. Associations between CSF rejuvenation protein levels and clinical outcomes are most consistent using a composite approach in adults with sporadic Alzheimer's disease. Effect sizes are standardized betas with 95% CI; CDR = Clinical Dementia Rating Scale global score. Estimates are displayed from multivariable regression models adjusting for age, sex, and education (n=91).

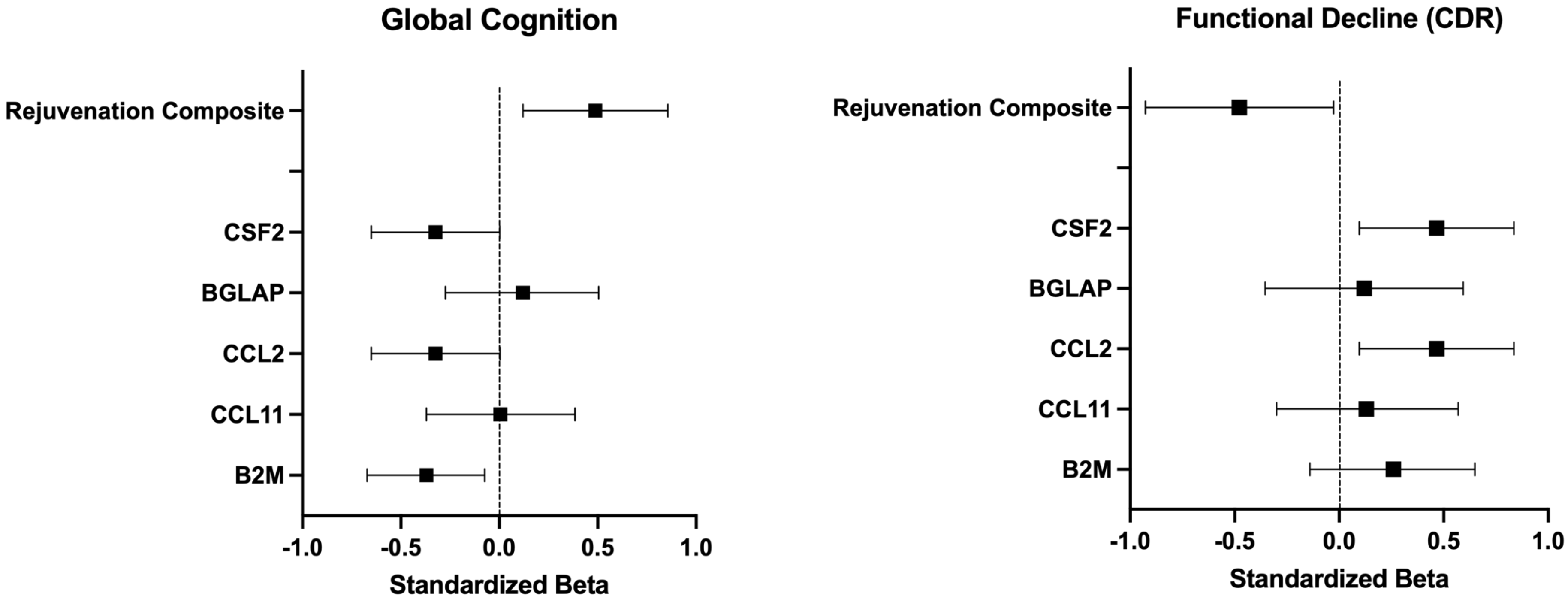

Supplement: fcae432_Supplementary_Data [file fcae432_supplementary_data.pdf]
